# Current Status of Obesity: Protective Role of Catechins

**DOI:** 10.3390/antiox12020474

**Published:** 2023-02-13

**Authors:** Tanisha Basu, Ashley Selman, Arubala P. Reddy, P. Hemachandra Reddy

**Affiliations:** 1 Department of Internal Medicine, Texas Tech University Health Sciences Center, Lubbock, TX 79430, USA; 2 Nutritional Sciences Department, College of Human Sciences, Texas Tech University, Lubbock, TX 79409, USA; 3 Neurology, Departments of School of Medicine, Texas Tech University Health Sciences Center, Lubbock, TX 79430, USA; 4 Public Health Department of Graduate School of Biomedical Sciences, Texas Tech University Health Sciences Center, Lubbock, TX 79430, USA; 5 Department of Speech, Language and Hearing Sciences, School Health Professions, Texas Tech University Health Sciences Center, Lubbock, TX 79430, USA

**Keywords:** obesity, mitochondrial dysfunction, hormonal deregulation, anti-inflammatory, antioxidant

## Abstract

Obesity is a growing health concern in today’s society. Current estimates indicate that obesity occurs in both adults and young people. Recent research also found that the Hispanic population in the U.S. is 1.9 times more likely to be overweight as compared to their non-Hispanic population. Obesity is a multifactorial disease that has a variety of causes. All current treatment options incorporate dietary changes aimed at establishing a negative energy balance. According to current scientific research, multiple factors are involved with the development of obesity, including genetic, biochemical, psychological, environmental, behavioral, and socio-demographic factors. The people who suffer from obesity are far more likely to suffer serious health problems, such as stroke, diabetes, lung disease, bone and joint disease, cancer, heart disease, neurological disorders, and poor mental health. Studies indicate that multiple cellular changes are implicated in the progression of obesity, mitochondrial dysfunction, deregulated microRNAs, inflammatory changes, hormonal deregulation, and others. This article highlights the role that oxidative stress plays in obesity and current obesity-prevention techniques with an emphasis on the impact of catechins to prevent and treat obesity.

## 1. Introduction

Obesity is a growing health concern in the modern world. According to the 2015–2016 National Health and Nutrition Examination Survey (NHANES), the average prevalence of obesity among adults in the United States (U.S.) was 39.8% with a prevalence of 35.7% among young people aged 20 to 40 years [1]. The prevalence of obesity has reached epidemic proportions during the previous five decades [1,2]. Between 1975 and 2016, the global prevalence of obesity increased threefold [3]. According to the World Health Organization (WHO), obesity afflicted an estimated 650 million people in 2016 [3]. Researchers found in 2022 that the Hispanic population in the Southeastern U.S. was 1.9 times more likely to be overweight as compared to their Caucasian counterparts [4]. As a result, obesity poses a huge public health risk. Obesity-related medical costs in the U.S. were estimated to reach $147 billion in 2008 [5]. 

Obesity is a multifactorial disease that has a variety of causes. All current treatment options incorporate dietary changes aimed at establishing a negative energy balance. According to current scientific research, genetic, biochemical, psychological, environmental, behavioral, and socio-demographic factors among others impact the development of obesity [5]. Those who suffer from obesity are far more likely to suffer serious health problems, such as stroke, diabetes, lung disease, bone and joint disease, cancer, heart disease, neurological disorders, and poor mental health [5,6].

The prevalence of childhood obesity is rising. Obesity in children aged 2 to 19 years of age increased from 4% in 1975 to 19.7% as of 2020 [3,7]. Hispanic children appear to be disproportionately affected with 26.2% Hispanic children as compared to 16.6% Caucasian children of the same age [7]. Childhood obesity is a serious health concern because children who are obese are five times more likely to become obese during adulthood [8]. 

Obesity is a chronic disease marked by the buildup of excess adipose tissue and is typically defined on the basis of body mass index (BMI), with a BMI of 30 or greater labeled as having obesity. There are three classes of obesity determined by BMI: class I (30–35 kg/m^2^), class II (35–40 kg/m^2^), and class III (40+ kg/m^2^). Childhood obesity is classified by having a BMI that is higher than 95% of those in their same age group after 2 years of age [9]. Obesity can then be further subdivided into central obesity (visceral) and subcutaneous obesity (more even distribution of fat under the skin). Centralized obesity poses a greater health risk than a more even fat distribution and is correlated to a higher risk of metabolic and cardiovascular disease [10].

Chronic inflammation is a hallmark of obesity and is directly linked to several health conditions seen by physicians. This inflammatory response leads to oxidative stress and damage linked to insulin resistance, high blood glucose and lipid levels, and hypertension which causes metabolic syndrome. The occurrence of metabolic syndrome is directly linked to type 2 diabetes mellitus (T2DM), cardiovascular disease, liver and kidney disease, sleep apnea, and cognitive decline [9]. This review article examines recent research on the role that oxidative stress plays in obesity and current obesity-prevention techniques with an emphasis on the impact of catechins to prevent and treat obesity.

## 2. Obesity: A Complex Multifactorial Disease

Obesity is defined physiologically as the formation of extra body fat (adiposity) caused by a long-term positive energy balance. Energy intake surpasses energy expenditure (EE) in a positive energy balance. The body then stores the excess energy as fat in adipose cells [1]. Obesity is traditionally thought to be caused by an increase in food consumption and a decrease in physical activity [5]. Dietary measures that induce a calorie deficit as well as physical activity guidelines are typically included in most treatment programs. Even though these treatments can help people lose 5 to 10 percent of their body weight in a short period, adherence rates are low and a success rates have a high rate of failure [2].

Researchers are attempting to explore and comprehend the various reasons for obesity. Obesity is linked to several complex internal (genetic and physiological) and external factors, such as socioeconomic and environmental factors [5]. This section summarizes current evidence and highlights individual risk factors, etiologies, and contributors to obesity, including oxidative stress, inflammation, and adipose tissue.

Individuals may have genetic abnormalities that affect neural and metabolic processes, resulting in obesity and/or a tendency to obesity when obesogenic environmental triggers are present [11]. Single gene mutations impact the core regulation of food and satiety via the gut-brain axis, resulting in monogenic obesity. The leptin receptors in the brain stimulate the pro-opiomelanocortin (POMC) neurons in the arcuate nucleus, resulting in a sequence of anorexigenic responses downstream [12]. Excessive eating and/or fat storage can be caused by mutations in any gene in this pathway [13]. Syndromic obesity, which is caused by certain prenatal diseases and neurodevelopmental anomalies, is another genetic kind of obesity. Among these are Bardet-Biedel Syndrome and Prader Willi Syndrome to name a few [14,15]. Polygenic obesity is caused by mutations in multiple genes that have a cumulative effect along with environmental factors. Scientists identified several of these genes and can be classified as body mass index (BMI)-related, overweight/obesity-related, or fat distribution-related [14]. Studies indicate that the genetic influence of some obesity genes is greater during childhood and non-genetic factors like household effects were most prominent during early adulthood [14]. The genetic effects of obesity have the least impact in older adults [15]. An area of growing interest among researchers is the investigation of how diet, lifestyle, and guy microbiome alternations can delay the onset and slow down the disease progression of genetic obesity [12,15].

Although evidence indicates that genetics plays a role in the pathogenesis of obesity. Research also suggests a strong role of various psycho-social factors that can act independently or further perpetuate the effects of these genetic factors [16]. Studies show a strong association of stress, depression, and addiction to eating with obesity. Another behavioral component that may contribute to obesity is the stigma present in society, media, and among healthcare providers. This stigma makes it difficult for individuals with obesity to seek care and reduces the rare adherence among those who do [2]. Negative emotional and cognitive effects are linked to such social stigmas, which further increases the rates of stress, depression, and food addiction [17].

**Figure 1:**
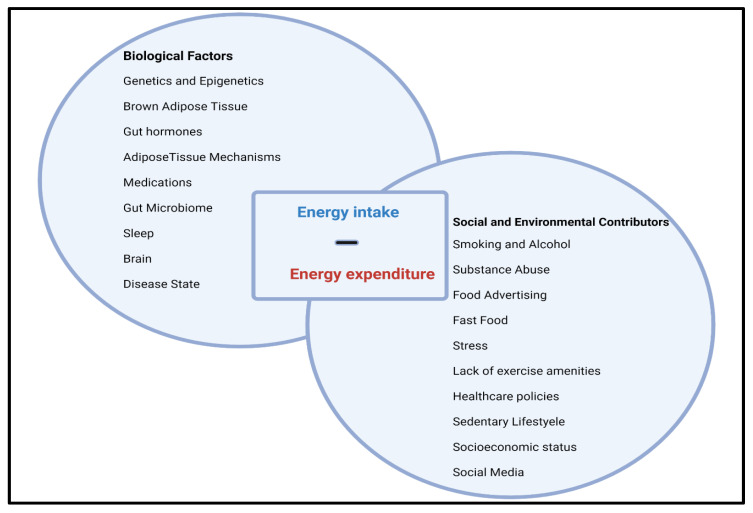
Obesity occurs when energy intake exceeds energy expenditure. This balance is a result of a multidetermined relationship between various biological factors, and social and environmental contributors.

Socioeconomic factors are also associated with obesity. Developed countries have a higher prevalence of obesity compared to developing countries. However, poverty has been positively correlated with the incidence of obesity in the United States (U.S.) over the past few decades [16]. Access to low-cost, energy-dense foods could be a reason for this [2,18]. Environmental factors like accessibility and affordability of healthy foods, presence of food desserts, the proximity of physical activity resources, time constraints, and nutrition awareness are all positively correlated with obesity (Figure 1) [18,19].

## 3. Obesity and Epigenetic Factors

Obesity is typically attributed to excessive caloric intake above the body’s energy demands. However, like many medial conditions the cause is multifactorial. Epigenetic modifications are one major contributing and predisposing factor. Epigenetics refers to alterations in gene expression due to methylation, acetylation and histone modifications to DNA that alters expression without changing the DNA sequence. These changes are heritable and can be modified by an individual’s lifestyle and choices. Thus, this is an important field in the study of obesity as some of these epigenetic modifications can predispose an individual to having obesity [20,21].

DNA methylation is the covalent attachment of a methyl group to a residue, particularly cytosines in the CpG sequence, within the genetic material [20,22]. Methylation is generally inactivating, leading to a decrease in gene expression. Heijmans et al. found that following a famine IGF-2 had lower rates of methylation to promote weight retention. This alteration would have been beneficial during times of low caloric intake; however, the subsequent six generations also were found to have the same epigenetic alteration. These individuals were found to have higher rates of obesity and cardiovascular disease [23,24].

Histone modifications are another key epigenetic alteration. Changes in DNA methylation can also impact histone modifications leading to histone deacetylation [20]. This deacetylation promotes further histone modifications, while furthering the inactive transcription state [20,24]. The most common alteration is acetylation at H3 and H4 lysine residues which promotes gene transcription. A HFD in obesity promotes histone acetylation of genes such as POMC and NPY, which regulate appetite and the dysregulation of which further perpetuates obesity [21].

The risk for developing obesity is not equal among all populations. Hispanic Americans are particularly at risk, with a 35% higher risk of developing obesity and developing subsequent chronic disorders [25,26]. Epigenetic mechanisms offer a potential explanation for this occurrence. Maternal obesity and gestational diabetes are critical risk factors because these conditions impact the uterine environment that has been associated with negative epigenetic alterations. This detrimental environment is believed to cause hypermethylation of the genes impacting metabolic regulation in the fetus and has a correlation with childhood and adult obesity [25]. Maternal obesity and gestational diabetes are more prevalent in minority groups such as Hispanics [26]. 

## 4. Oxidative Stress and Inflammation

Many studies link obesity to persistent oxidative stress. The imbalance between the accumulation and generation of reactive oxygen species (ROS) causes oxidative damage to cells and tissues [27,28]. ROS are natural byproducts of oxygen metabolism and are important regulators of various physiological functions, including cell signaling [29]. Environmental stressors (e.g., ultraviolet light, ionizing radiation, pollution, heavy metals) produce an imbalance in ROS production which causes cell and tissue damage [29].

Mitochondria produce hydrogen peroxide (H_2_O_2_), superoxide radicals (O_2_), hydroxyl radicals (OH), and singlet oxygen (1O_2_) [27,30]. Protein phosphorylation, activation of many transcriptional factors, apoptosis, immunity, and differentiation all produce ROS as a metabolic consequence of mitochondrial and cellular processes [31,32]. The mitochondrial respiratory chain and the following oxidase enzymes are the principal producers of ROS: nicotinamide adenine dinucleotide phosphate (NADPH) oxidases, xanthine oxidases (XO), lipoxygenases, cyclooxygenases, cytochrome P450 enzymes, and uncoupled nitric oxide synthases [33]. For example, enzymes like lipoxygenases (LOX) and cyclooxygenases (COX) in endothelial and inflammatory cells generate ROS during arachidonic acid metabolism. When kept at low or moderate levels, free radicals have several beneficial effects on the body. ROS are required for the synthesis of several cellular structures and for the host immune system to release free radicals to the invading pathogens and microbes [29,32,34]. 

Dysregulated ROS generation in cells can produce oxidative stress by causing negative effects on key cellular components (e.g., proteins, lipids, nucleic acids) [35,36]. When the formation of ROS exceeds the antioxidant defense, oxidative stress ensues [37]. Examples of negative impacts are: (1) lipid peroxidation that damage membrane lipids, and (2) conformational modification of proteins that reduce enzymatic activity, create deoxyribonucleic acid (DNA) lesions that promote mutagenesis [37,38]. Extensive data indicates that oxidative stress may have a role in the development of diabetes, metabolic disorders, atherosclerosis, cardiovascular diseases, and cancer in varying degrees.

## 5. Oxidative Stress and Obesity

Obesity and oxidative stress are deeply intertwined [37]. Obesity is typically caused by caloric overconsumption and a lack of physical activity [39]. As a caloric reserve, adipose tissue expands to tolerate overnutrition, resulting in maladaptive remodeling [40]. Adipokines, which are bioactive molecules released by adipose tissue, play a crucial role in regulating systemic metabolism and inflammation. Obesity-induced adipose tissue dysfunction changes the secretion patterns of adipokines, which modulates distant tissues such as the cardiovascular system [41]. The enlargement of adipose tissue leads to ectopic fat deposition in other organs (e.g., liver, heart, kidney) which worsens metabolic problems [27,42]. When the formation of reactive oxygen species (ROS) exceeds the antioxidant defenses, oxidative stress ensues [41]. The irregular production of ROS and oxidative stress in adipose tissue can result in a variety of pathophysiological situations. As a result, oxidative stress in adipose tissues may be targeted for obesity prevention and therapy [41].

### 5.1. Role of Mitochondrial Dysfunction in Obesity

Mitochondria are responsible for the creation of the human body’s energy currency and adenosine triphosphate (ATP) from food macromolecules and houses the majority of the cellular pathways [43]. The process of cellular respiration is an essential part of metabolism. Although this process yields large amounts of ATP, it also generates free radicals that lead to oxidative stress [44]. However, these free radicals are generally balanced by an antioxidant defense in an ideal situation. This defense is made up of enzymes, such as superoxide dismutase (SOD), catalase, and glutathione peroxidase (GP) [44]. 

### 5.2. Obesity: An Unbalanced Metabolic State

A surplus caloric intake overwhelms the enzymatic defense within the mitochondria which allows free radical generation to go unchecked and causes mitochondrial dysfunction [43,45]. The Bonnard et al. mouse model illustrated that a high-fat diet (HFD) rich in sucrose caused an increase in the amount of reactive oxidative stress (ROS) and mitochondrial alterations in the muscle tissue of the mice [46]. Mitochondrial dysfunction is defined in two ways: (1) an inability of the mitochondria to meet the ATP requirements of the cell and (2) a maladaptive change in the mitochondrial function that perpetuates metabolic syndrome [44,47]. These detrimental adaptations to a HFD and nutrient excess in adipocytes leads to a decrease in mitochondrial biogenesis and the rate of β-oxidation which contributes to insulin resistance, another key hallmark of metabolic syndrome [44]. This increase in ROS mediated by mitochondrial dysfunction and insulin resistance further adds to the inflammatory state correlated with obesity.

### 5.3. Adipose Tissue and Oxidative Stress

Adipocytes are specialized cells that make up adipose tissue, which is made up of loose connective tissue [48]. Adipocytes are divided into two types: (1) energy-storing white adipose tissue (WAT) and (2) heat-storing brown adipose tissue (BAT). These two types of tissues account for 4.3 percent of total fat mass [49]. Brown adipocytes have multiple, small lipid droplets and a plethora of mitochondria, which gives BAT a brown color and a multilocular histological appearance [50,51]. Adipose tissue can further be classified based on the anatomical localizations as either: (1) subcutaneous adipose tissue (SAT) or (2) visceral adipose tissue (VAT) [51]. This distinction is important as VAT is more closely linked to metabolic syndrome. VAT releases a different hormone profile that SAT. The biomolecules released by include adiponectin, leptin, and key inflammatory cytokines (i.e., TNF-α, IL-6), which all contribute to the development of metabolic syndrome [48,52].

Other cells found in adipose tissue include preadipocytes, fibroblasts, vascular endothelial cells, and a variety of immune cells (e.g., adipose tissue macrophages) [34,35]. Excess energy is stored as triglycerides in lipid droplets in adipose tissue; ingested fatty acids are simply esterified. Adipose tissue cushions and insulates the body and serves as a primary source of hormones, such as leptin, estrogen, resistin, and proinflammatory cytokines like tumor necrosis factor alpha (TNF-α) [37]. These hormones are all contributors to the maintenance of metabolism and excessive amounts of adipose skews the levels of these molecules, impacting the development of obesity and metabolic syndrome. Adiponectin is responsible for sensitizing the body’s tissues to insulin, but large amounts of adipose tissue counterintuitively leads to a lower level of circulating adiponectin and perpetuates insulin resistance [48]. Resistin also has a hand in the development of insulin resistance due to its’ role in interfering with insulin action. Along with these hormones promoting insulin resistance and a state of obesity, adipose tissue also creates an inflammatory environment. This low-grade, chronic inflammation is due to macrophages present. Weisberg et al. demonstrated that macrophages can account for 60% of the tissue’s makeup [53]. These macrophages are responsible for releasing important inflammatory cytokines and contributing to the ROS and increasing the capacity for oxidative stress [44,48].

The regulation of obesity-induced oxidative stress in adipose tissue is depot-specific [54,55]. According to several human and rodent investigations that compared subcutaneous adipose tissue (SAT), oxidized lipids and proteins accumulate in visceral adipose tissue (VAT) [56,57,58]. Multiple research studies on lipid peroxidation, which is one of the hallmarks of oxidative stress, found that these products are regulated differentially in SAT. Although lipid peroxidation was higher in epididymal adipose tissue (EAT) and lower in SAT in both high-fat diet (HFD)-induced obese mice and ob/ob animals, exercise training-mediated oxidative stress reduction lowered lipid peroxidation and NADPH oxidase expression in SAT, but not VAT [59,60].

Research indicates that subjects with obesity have increased adipose tissue, mitochondrial oxidative stress indicators (e.g., protein carbonyls, lipid peroxidation products, malondialdehyde [MDA]), and increased reactive oxidative stress (ROS) production [32]. Scientists found enhanced generation of ROS in adipocytes and a buildup of the lipid peroxidation product 4-hydroxynonenal (4-HNE) in db/db mice [61]. Obesity-related oxidative stress can be driven by some factors, notably poor nutritional status, hyperglycemia, hyperlipidemia, and chronic inflammation [62].

**Figure 2:**
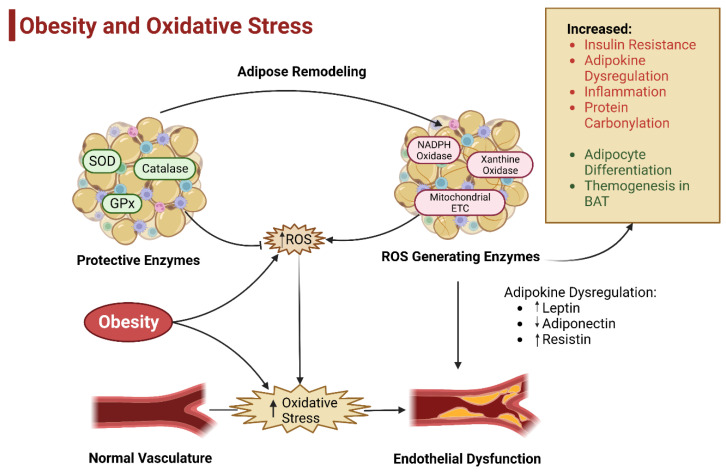
Oxidative Stress and Obesity. The body is greatly impacted by the balance between antioxidants and protective enzymes against the amount of ROS generated. Obesity leads to an increase in the production of ROS and leads to an increase in oxidative stress which leads to adipokine dysregulation and vascular damage, hyperinsulinemia, and the inflammatory state (adapted from Zhou et al. 2021) [37].

Studies indicate that subjects with obesity who eat large meals which contain about 1800 kcal and contain high amounts of fat and carbohydrate have increased ROS production by mononuclear cells [63]. Furthermore, subjects with overweight and obesity who lack consumption of protective antioxidant phytochemicals in their diet show a decline in plasma levels of vitamins and minerals and an increase in oxidative stress [33,64]. Obesity causes a quantitative and qualitative alteration in adipose tissue cellular composition, which would be accompanied by adipose tissue expansion. Of these cellular changes, an increase in the inflammatory response is the most detrimental. Macrophages are the most abundant immune cells in adipose tissue and are key mediators of the inflammatory response. The recruitment and proliferation of macrophages under high-fat diet conditions are directly linked to adipose tissue inflammation. There are two distinct types of macrophages: (1) pro-inflammatory M1 and (2) anti-inflammatory M2. Pro-inflammatory cytokines are released by activated M1 macrophages. These inflammatory cytokines include tumor necrosis factor-alpha (TNF-α), interleukin (IL)-6, and IL-1, all of which promote ROS generation in adipose tissue (Figure 2) [37,57]. 

Obesity is associated with low-grade chronic inflammation due to the cellular inflammatory components of adipose tissue, which serves as a major source of oxidative stress [65]. Although adipocytes make up the majority of adipose tissue volume, the tissue also comprises stromal vascular fractions (SVFs). Examples of SVFs include: preadipocytes, fibroblasts, vascular endothelial cells, and immunological cells [58]. TNF- treatment of 3T3-L1 adipocytes reduced the expression of mitochondrial antioxidants such as glutathione S-transferase A4 (Gsta4), peroxiredoxin 3 (Prx3), and glutathione peroxidases (GPx), resulting in increased protein carbonylation, ROS generation, and mitochondrial dysfunction [60]. Mice without the myeloid cell-specific NADPH oxidase 2 (Nox2) genes had a protective effect against adipose tissue inflammation produced by a high-fat diet (HFD) and better metabolic functioning [66].

#### 5.3.1. Sources of ROS in Adipose Tissue

Nox is a multicomponent enzyme that produces reactive oxygen species (ROS) when it transfers electrons from nicotinamide adenine dinucleotide phosphate (NADPH) to oxygen across the cell membrane. There are seven isoforms in the Nox family: Nox1, Nox2, Nox3, Nox4, Nox5, Duo1, and Duo2 [67]. Nox4 is the sole isoform of the Nox family that is expressed in adipocytes [55]. Scientists transiently elevated adipocyte Nox4 and pentose phosphate pathway activity in mice during obesity development driven by a high-fat and high-sucrose diet to successfully prevent obesity-induced insulin resistance [68].

Researchers derived primary adipocytes from Nox4 mutant mice resistant to inflammation produced by high glucose or palmitate concentrations. Researchers administered a Nox inhibitor to activate free fatty acids (FFAs) to increase oxidative stress in cultured adipocytes [69]. In obese mice models, the inhibition of NADPH oxidase significantly reduced reactive oxygen species (ROS) generation in the white adipose tissue (WAT) and alleviated obesity-induced adipose tissue dysfunction [69]. This study identified the generation of ROS by mitochondria as the primary cause of oxidative stress in adipose tissue. Mitochondria create energy by oxidative phosphorylation, which produces (ROS). The electron transport chain’s (ETC’s) complexes I and III are the primary sources of ROS generation [70]. Excess nutrients in adipocytes increase mitochondrial substrate loading in obesity, leading to increased ROS production in mitochondria [71]. Other in vitro experiments showed that high glucose or FFA concentration increased ROS production in mitochondria [72,73].

The xanthine dehydrogenase (XDH)/oxidoreductase (XOD) system is another enzyme system associated with adipocyte ROS production. XOD predominates in the form of XDH under normal conditions, but transforms to XO (the predominant source of ROS generation) during oxidative stress. Purine bases are converted to uric acid by XO, an oxidant form of XOD [74]. A clinical trial found obesity to be an independent predictor of elevated XO activity in a clinical trial of volunteers with overweight and obesity [74]. ROS, such as ^1^O_2_ and H_2_O_2_, may be linked to de novo lipogenesis and ROS generation [57,58]. 

#### 5.3.2. Effect of ROS in Adipose Tissue

The oxidative stress caused by obesity in adipose tissue is a primary factor linked to cellular malfunction and insulin resistance [68]. Studies found that insulin-induced activation of glucose transporter type 4 (GLUT4) decreased in 3T3-L1 adipocytes after prolonged exposure to H_2_O_2_ [75]. Scientists discovered that reactive oxygen species (ROS) generation is linked to the dysregulation of adipokine expression in adipose tissues in obesity [38,59].

The adipose tissue oxidative stress-activated nuclear factor kappa-light-chain-enhancer of activated B cells (NF-kB) and mitogen-activated protein kinase (MAPK) downregulates anti-inflammatory adipokines and stimulates pro-inflammatory cytokines. Increased oxidative stress-induced adipocytokine production to be dysregulated in cultured adipocytes, including adiponectin, IL-6, and monocyte chemotactic protein 1 (MCP1) [66,69]. In adipose tissue, a prominent symptom of oxidative stress is protein carbonylation or the irreversible modification of proteins by reactive lipid aldehydes. The most widely studied aldehyde products of lipid peroxidation are 4-HNE and 4-oxonononenal (4-ONE), which are ubiquitous in adipose tissue [65]. Scientists elevated the amounts of lipid peroxidation products in the epididymal adipose tissues (EAT) of murine models 5- to 11-fold. Scientists potentiated 4-HNE carbonylation of histones within adipose tissue of ob/ob mice and high-fat diet (HFD)-induced obese mice [74].

Although ROS are frequently linked to cardiovascular disease and poor metabolic outcomes, they also serve a crucial regulatory role in adipose tissue biology. Lee et al. (2009) found the elevation of peroxisome proliferator-activated receptor gamma (PPAR) expression accelerated 3T3-L1 cell differentiation after H_2_O_2_ treatment [72]. Chouchani (2016) determined acute thermogenesis activation in brown adipose tissue (BAT) significantly increases mitochondrial ROS, but reduced pharmacological treatment reduced mitochondrial ROS and caused hypothermia in response to cold exposure and prevented the uncoupling protein 1 (UCP1)-dependent increase in whole body energy expenditure (EE) [76]. 

## 6. Endogenous Antioxidants

Research indicates that a high-fat diet (HFD) causes catalase to inhibit lipogenesis and Nox4 expression in adipocytes which limits weight gain and fat mass gain [68]. Huh et al. (2012) found that catalase and superoxide dismutase (SOD)-1 overexpression in adipocytes can help avoid ectopic fat accumulation and improve insulin sensitivity [77]. Other studies indicate that heme oxygenase 1 (HO1) can enhance adiponectin expression and reduce hyperglycemia and insulinemia in female mice and increase vascular function and insulin sensitivity and that Prx-2 increases adipogenesis and Prx3 promotes adiponectin expression [37,78].

Studies indicate the depletion of these antioxidant enzymes in adipocytes has detrimental effects on adipocyte functions and promotes the development of cardiometabolic diseases. In addition, glutamate-cysteine ligase (Gclc) facilitates glutathione synthesis and inhibits ROS production, thereby inhibiting ectopic fat accumulation and insulin resistance. However, deletion of either SOD2 or glutathione peroxidases (GPx) reportedly provides beneficial effects in adipose tissue function [37,79]. The anti-obesity effect of SOD2 deletion in adipocytes can be attributed to activated mitochondrial biogenesis and enhanced mitochondrial fatty acid oxidation, which can promote energy expenditure (EE). Insulin signaling can be enhanced by knocking down GPx in either muscle cells or hepatocytes; GPx-1 deletion can attenuate inflammation and enhance browning in visceral adipose tissues (VATs) [79,80].

### 6.1. Dietary and Pharmacological Strategies

According to epidemiological studies, the obesity epidemic and the comorbidities that come with it continue to be a global health issue [81]. Obesity is more common in individuals who consume a Western diet (WD), which is deficient in several nutrients and high in fat (30–40% of kcal in diet) [82]. The WD also stimulates the body’s inflammatory response which contributes to cellular damage [83]. Research during the past decade helped develop dietary, pharmacological, and surgical strategies to mitigate the metabolic effects of a high-fat diet (HFD) [82]. Although pharmacological and surgical interventions are often more effective at preventing obesity, high costs and life-threatening side effects are still potential downsides. Nutritional alteration and exercise incorporation may be the safest and most cost-effective option for those who are moderately obese, and it is still the primary intervention used today [84]. Natural products (e.g., crude extracts, compounds extracted from plants) can help with weight loss and prevent diet-induced obesity and are now increasingly employed in the treatment of obesity [55,85].

### 6.2. Protective Role of Quercetin in Obesity

Quercetin is a flavonoid that is naturally found in plants, fruits and vegetables and studied for its antioxidant therapeutic properties [86]. In a study, it was demonstrated that quercetin inhibited lipid buildup and obesity-induced inflammation. Differentiated adipocytes after quercetin treatment exhibited suppressed expression of C/EBPβ, an early adipogenic factor, and thereafter C/EBPα, PPARγ and FABP4, key adipogenic factors. Animal studies have discovered that quercetin can shield mice or rats from high-fat diet- (HFD-) induced excess adiposity leading to obesity [87,88,89]. In HFD-fed mouse model, Stewart et al. explored that quercetin can briefly upturn energy expenditures which may be associated with upregulation of UCP-1 (UnCoupling Protein-1) [89]. In the same model, quercetin has shown results to block the process of adipogenesis directly acting on the adipogenic factor C/EBP α gene expression levels and decrease lipogenesis by downregulating the gene levels of FAS and ACC [86,90]. Quercetin also possesses anti-inflammatory effects on adipose tissue and long-term supplementation with quercetin can help reduce inflammatory markers IFNγ, TNFα, IL-1, and IL-4 in mice. Quercetin increases the levels of oxidative stress sensitive transcription factor and promotes mitochondrial function by limiting immune cell activation in adipose tissue of HFD-induced obese mice [86,91].

While various animal studies have demonstrated the role of quercetin in obesity, very few clinical trials have done the same. In a 12-week, randomized, double-blind, placebo-controlled study, 100 mg/day of quercetin significantly decreased the total body fat percentage and decreased the body mass index (BMI) of subjects with overweight or obesity [92]. In another study the effect of quercetin on obesity with subjects who had various apolipoprotein E (APOE) genotypes were investigated. It was seen that 150 mg/day of quercetin decreased the waist circumference and triacylglycerol concentrations [86,93]. Additionally, one study demonstrated that a 12-week intervention of quercetin rich onion extract intake decreased body weight, body fat percentage, and BMI of 10 female university students. However, another study contradicted these results with no significant changes reported in body composition after 12 weeks of onion extract supplementation [86,92,94,95]. It was reported that quercetin has no effect on oxidative stress and antioxidant capacity in subjects with overweight and obesity who consumed a high dose (500 or 1000 mg/day) of quercetin during a 12-week period [86,96]. Future researchers need to further investigate the pharmacological effects and bioavailability of quercetin in the treatment of obesity in clinical trials.

### 6.3. Protective Role of Curcumin in Obesity

Current studies indicate that curcumin, the bioactive polyphenol in the spice turmeric, has beneficial effects on body weight reduction and energy metabolism [97,98]. In rats fed two weeks of high doses of curcumin, there was a reduction in epididymal adipose tissue, increase in fatty acid β-oxidation, and an overall increase of energy expenditure [98,99]. Dietary curcumin dosed at 0.2–1 g/100 g diet was also seen to reduce lipid accumulation in epididymal adipose tissue [99]. In obesity mice models, both HFD-induced and genetic (ob/ob), curcumin was shown to reduce inflammation in the adipose tissue by limiting the rate of infiltration by macrophage into adipose tissue and promoting adiponectin production [98,100,101].

Unlike studies on the effects of curcumin in cells or animals, studies on human subjects with obesity are limited [98,102]. Mohammadi et al. conducted one of the first clinical trials using curcumin for obesity treatment in which subjects with obesity were treated with a commercially available version of curcumin, C3 Complex^®^, (1 g/day) over a period of one month. They were also given a bioavailability enhancer, piperine (5 mg/day). There were no significant changes in body weight or body composition, however, there was a significant drop in serum triglycerides [98,103]. Similarly, in another randomized, double-blind, crossover trial, a 30-day supplementation schedule of C3 Complex (500 mg/day) along with piperine (5 mg/day) significantly reduced levels of inflammatory cytokines IL-1β and IL-4 in subjects with obesity, indicating the potential anti-inflammatory role of curcumin in obesity treatment [104]. Moreover, 1 g/day for 30 days of oral curcumin supplementation effectively reduced oxidative stress in individuals with overweight and obesity [98,105]. The breadth of pharmacokinetics and pharmacological effects of curcumin in humans is not fully known and warrants further investigation. According to World Health Organization and the Food and Agriculture Organization, the maximum daily dose of curcumin is 1 mg/kg of body weight [98]. However, a few studies have indicated liver toxicity to be an outcome of prolonged curcumin use. Moreover, high doses of curcumin can cause side effects such as gastrointestinal distress, skin inflammation and chest tightness [106,107].

### 6.4. Polyphenols and Catechins

Green tea, which is derived from the tea plant (*Camellia sinensis*), is the world’s second most popular beverage and is high in polyphenols. Scientists have thoroughly examined green tea and its polyphenols for its health-promoting potential for the last decade. Polyphenols, including catechins (flavanols), anthocyanins and leucoanthocyanidins, phenolic acid, depside, flavonoids, and flavanols, constitute approximately 18–36% of the dry leaf content in green tea [108].

**Figure 3:**
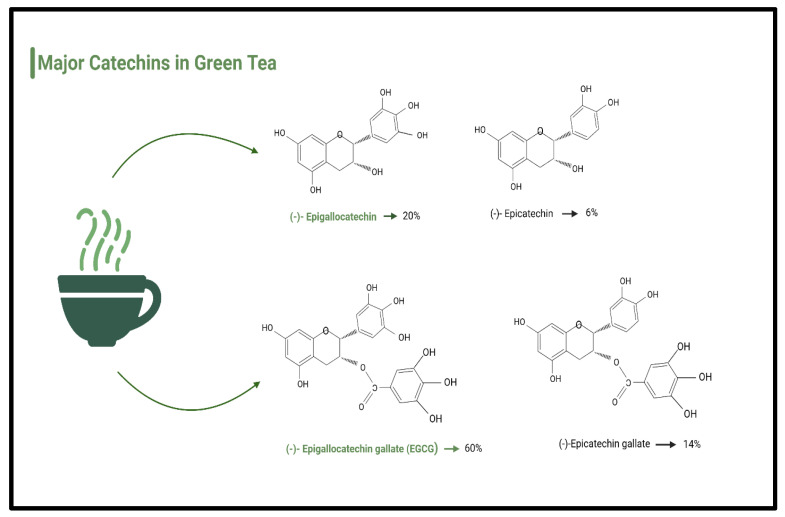
Types and percentages of major catechins in green tea.

According to Isemura (2019), the three most prevalent catechins brewed from green tea include the following: (1) epigallocatechin (EGC)—13.08%, (2) epicatechin gallate (ECG)—15.44%, and (3) epigallocatechin gallate (EGCG)—60.89% [108] (Figure 3). Most research on green tea focuses on its anti-cancer properties. Therefore, scientists are currently investigating putative molecular mechanisms of action of anti-tumorigenic activity [109]. Catechins may also have a range of additional therapeutic qualities, notably anti-inflammatory, anti-arthritic, anti-bacterial, anti-angiogenic, anti-oxidative, anti-viral, and neuroprotective properties in Parkinson’s disease (PD) [110,111,112]. 

#### 6.4.1. Protective Role of Catechins in Obesity: Mechanisms of Action

The use of green tea is growing in popularity among molecular nutritionists and food experts as a treatment for animals and humans [113]. Numerous studies are underway to investigate the effect of catechins in green tea on hyperlipidemia and fat mass gain in obese rodent models fed a high-fat diet (HFD). However, the exact anti-obesity effects of green tea in humans, as well as the underlying signaling pathways that regulate body weight management, are unknown [113].

Studies indicate that catechins have an impact on neuroendocrine metabolic regulators of appetite and thereby decrease food intake [113]. They are also associated with reduction of the process of emulsion and absorption of lipids and protein in gastric tract and therefore diminish calorie consumption [114,115]. Interestingly, catechins have presented an effect on gastrointestinal microbiota (lacto- and bifidobacteria), which are accountable for the process of food digestion [113]. They yield short fatty acids, which enhance the speed of lipid metabolism [113,115]. Studies have also shown that they restrict the differentiation and proliferation of preadipocytes [113]. Collectively, they reduce lipid production, promote lipolysis and lipid metabolism [113,115]. Catechins arouse transformation of white adipose tissue to brown, escalate its oxidation, burning and expenditure of energy through heat generation, and promote fecal lipid excretion [113,115]. An animal study showed that reduced digestibility and a rise in energy expenditure and fat oxidation via β-adrenoceptor activated thermogenesis of brown adipose tissue are all processes which are initiated and conducted by catechins present in green tea [116]. One major action of tea constituents occurs in the gastrointestinal tract by diminishing digestion activity and absorption of macronutrients, or by modifying the gut microbiota. The other type of action is inhibition of anabolism and stimulation of catabolism in liver, muscle, adipose tissue [113]. Anticipated mechanisms for the actions of tea constituents in lowering body weight could include the activation (phosphorylation) of AMPK regulates metabolism in different organs which downregulates gluconeogenesis, fatty acid synthesis, insulin secretion and ectopic fat deposition in muscle and liver. These processes are supplemented by an increase in insulin sensitivity and the oxidation of glucose and fatty acids [117,118].

#### 6.4.2. Protective Role of Catechins in Obesity: Animal Models

In rodents fed high-fat diets (HFDs) or genetically obese/diabetic animal models, consumption of green tea extract or epigallocatechin gallate (EGCG) significantly: (1) reduced body weight and/or adipose tissue, (2) decreased blood glucose or insulin levels, and (3) increased insulin sensitivity or glucose tolerance (Table 1). One study found that when compared to the HFD-only group, EGCG treatment significantly reduced body weight gain, attenuated insulin resistance, and reduced blood glucose and liver TAG levels in obese mice induced by a high-fat/Western diet (HF/WD) [119]. Additionally, db/db mice that received dietary supplementation with EGCG (1 percent of diet) resulted in diminished body weight and reversed the advancement of glucose intolerance [120]. Similarly, another study observed that feeding mice dietary EGCG (3.2 g/kg diet) for 16 weeks reduced body weight gain, body fat percentage, and visceral fat weight as compared to mice that did not receive EGCG therapy [92,121]. In another study, C57BL/6J mice received a HFD for 8 weeks to induce obesity and then separated into three groups and maintained either on a HFD-controlled diet or an HFD supplemented with 0.2 or 0.5 percent EGCG (*w*/*w*) for another 8 weeks. EGCG dramatically decreased body weight, various adipose tissues, plasma triglycerides (TAGs), and liver lipids [72].

In vitro studies show that catechin-enriched green tea extracts could increase sympathetic-mediated thermogenesis in brown adipose tissue (BAT) [21,124]. Scientists found that green tea extracts boost energy expenditure in mice via stimulating brown fat thermogenesis [96]. Tea catechins seem to have an anti-obesity effect in mice fed a HFD and stimulated lipid catabolism in the liver [125,126]. In mice, dietary EGCG attenuated diet-induced body fat storage [127]. Although EGCG enhanced fat oxidation, its fat-burning impact could be explained entirely by its impact on food digestibility [100]. In a separate trial, mice were given 50 mg/kg and 100 mg/kg each day, along with a HFD for 20 weeks. EGCG reduced obesity, weight of epididymal adipose tissue, and blood lipid characteristics, such as triglyceride, cholesterol (CHOL), and high- and low-density lipoprotein CHOL (HDL-C, LDL-C) concentrations [101]. In both the HFD and EGCG groups, expression of genes involved in the synthesis of de novo fatty acids (i.e., acc1, fas, scd1, c/ebp, ppar, and srebp1) decreased and expression of genes involved in lipolysis (hsl) and peroxidation in white adipose tissue increased. Results included elevated AMPK activity in both subcutaneous adipose tissue (SAT) and epididymal adipose tissue (EAT) indicating that EGCG can partially decrease obesity and white EAT weight in mice via AMPK activation [128].

#### 6.4.3. Catechins in Obesity: Human Studies

Epigallocatechin gallate (EGCG) and epigallocatechin (EGC) are the types of catechins most abundantly present in brewed green tea. Scientists are currently investigating green tea and its extract (catechin), catechin-rich teas, and other sources of catechins to: (1) enhance energy expenditure (EE), (2) increase fatty acid oxidation and thermogenesis, and (3) reduce fat absorption. Scientists also propose that tea polyphenols may counteract the decrease in resting metabolic rate (RMR) associated with weight loss [113,129] (Table 2). Studies show that catechin along with caffeine can reduce body weight and waist circumference by having a synergistic effect on adipose tissue thermogenesis [113,130]. Catechins also reportedly prevent adipogenesis in mature adipocytes, prevent the differentiation of preadipocytes to adipocytes, and therefore, reduce fat accumulation in adipose tissues [131]. Catechins are also positively associated with improvements in biomarkers like insulin, glucose, high-density lipoproteins (HDL), low-density lipoproteins (LDL), and total cholesterol. Studies indicate the consumption of catechin-rich teas correlate to increased endurance capacity and exercise tolerance which may possibly boost EE [21]. Scientists also report catechins have potential benefits in sleep regulation which in the past decade were examined as a potential contributor to body weight gain [129]. 

Clinical studies investigating the anti-obesity properties of catechins show promising results. An uncontrolled study in overweight individuals found a 3.5 kg mean weight loss and 4.14 cm decrease in waist circumference from baseline to completion [113]. Although results were significant, the lack of a case-controlled design make them less valid. However, other studies that used randomized controlled trial (RCT) designs did yield similar results. A single-blind, RCT conducted in individuals with obesity found a significant decrease in body weight and waist circumference over 8 weeks of supplementation with a green tea beverage that contained EGCG and caffeine as compared to a placebo beverage [132].

Katada et al. recently conducted a double-blind, randomized, placebo-controlled, crossover study in 16 middle-aged men and 10 women to examine the effects of catechin with caffeine on EE. Scientists took baseline measurements, measured fasting, resting metabolic rate (RMR) and EE after the 2-week ingestion of test beverage, and simultaneously measured forehead temperature (a proxy for core temperature) and skin temperature. Results indicated that EE increased significantly after ingestion of the tea catechin beverage compared with the placebo beverage [132]. Katada et al. concluded that ingestion of tea catechin along with caffeine for 2 weeks increases thermogenesis and EE immediately after ingestion of the test beverage. The study design was strong because the amount of caffeine was matched between the test and placebo beverages. Therefore, the effects observed can be attributed to the catechin present in the test beverage [132]. However, the study conclusion is limited because this trial did not investigate the mechanism of action. 

Another randomized, double-blind study conducted by Chen et al. examined the effects of high-dose green tea extract (EGCG) at a daily dose of 856.8 mg on weight loss, lipid profile, and hormone peptides in women with central obesity. This study included 102 women with BMI ≥ 27 kg/m^2^ and a waist circumference (WC) ≥ 80 cm who were randomly assigned to green tea or placebo intervention for 12 weeks. Results indicated significant weight loss, from 76.8 ± 11.3 kg to 75.7 ± 11.5 kg (*p* = 0.025), as well as decreases in BMI (*p* = 0.018) and waist circumference (*p* = 0.023) in the treatment group after 12 weeks. Significantly lower ghrelin levels and elevated adiponectin levels were detected in the study group than in the placebo group. This study also demonstrated a consistent trend of decreased total cholesterol (reaching 5.33%) and decreased low-density lipoprotein (LDL) plasma levels [130]. Huang et al. conducted a similar randomized, double-blind, placebo-controlled, crossover study conducted to investigate the effects of a 6-week green tea extract intervention on 90 women who had overweight/obesity with high LDL cholesterol as compared to a placebo (cellulose). Data from 73 participants who were analyzed indicated a significant between-group difference in LDL cholesterol (*p* = 0.048) and leptin (0.046) [102]. Results of these studies are strong due to the utilization of double-blinded randomized control trials (RCTs).

Another novel study conducted by Most et al. attempted to investigate the effects of polyphenols, specifically EGCG with resveratrol (EGCG + RES) on adipose tissue morphology and gene expression. The study utilized a randomized, double-blind, placebo-controlled trial of 38 men and premenopausal women who had overweight/obesity. Adipose tissue biopsies were obtained for 25 participants. Results indicated that pathways related to oxidative stress, inflammation, and the immune response showed lower expression levels in adipose tissue after intervention with EGCG + RES [133].

## 7. Conclusions: Gaps in Research and Future Directions

Plant polyphenols, including catechins found in tea, have antioxidant properties that can reduce reactive oxidative stress (ROS) populations and reduce inflammation in people afflicted by obesity. Various mouse studies indicate that there are two major mechanisms involving epigallocatechin gallate (EGCG): (1) decreased absorption of lipids and proteins in the intestine that reduce calorie intake and (2) activation of AMP-activated protein kinase (AMPK) in the liver, skeletal muscle, and white adipose tissue (WAT). According to the “AMPK hypothesis,” AMPK plays a major role in mediating the actions of EGCG on fatty acid synthesis and fatty acid catabolism. Only one study reports activation of AMPK by EGCG in cultured cells and liver tissues in mice, which suggests the effects of catechins (including anti-obesity and anti-cancer effects) are partially mediated by activation of AMPK [134]. Therefore, it is still unclear whether AMPK activation plays a key part in EGCG—induced adipose tissue reduction or performs an equivalent activity in numerous depots [113].

Uncontrolled clinical studies with green tea catechin supplementation (the equivalent of 3–4 cups daily) demonstrated favorable changes in body weight and body fat between baseline and completion. However, scientists find no significant differences when compared to a control group [113]. Studies using randomized, blind, and case-controlled trials found significant differences in body weight, energy expenditure (EE), body mass index (BMI), waist circumference, lipid markers, and gut hormones [113,122,129,130,132]. However, most of these studies involved small sample sizes, gender-specific data, use of catechins with other polyphenols or caffeine, and short intervention periods. The dose, type, and method of delivery (beverage or capsules) of catechins used in the studies were heterogeneous. Therefore, there is a lack of human clinical research to demonstrate the therapeutic benefits of catechins in obesity prevention and management and to elucidate the mechanisms of action through which catechins might aid weight loss or weight management. The discrepancies in human studies indicate a gap in knowledge of the specific target populations, dietary patterns, ethnic phenotypes, and other demographic characteristics that can determine the efficacy of catechins as a weight loss intervention. The bioavailability of catechins and their metabolic breakdown need to be further investigated to truly understand its anti-obesity properties and use in clinical recommendations [129]. The role of catechins in brown adipose tissue (BAT) thermogenesis and subsequent energy expenditure (EE) in rodents should be studied in human models. Randomized clinical trials (RCTs) need to be conducted in diverse populations with specific doses and types of catechins to assess their effects on body weight, body composition, blood biomarkers, inflammatory markers, adipose tissue morphology, and other metabolic markers to truly understand its clinical ramifications in obesity treatment.

## Figures and Tables

**Table 1:** Catechin and Antioxidant Studies in Animal Models.

Study Name	Author and Year	Methods	Result
Green tea, black tea, and epigallocatechin modify body composition, improve glucose tolerance, and differentially alter metabolic gene expression in rats fed a high-fat diet [119]	Chen et al., 2009	Rat models were fed a HFD for 6 months and were treated with either: EGCG, GT, BT or water control.	GT, BT, and EGCG all improved glocuse tolerance as compared to the control. GT, BT increased FA oxidation, but not EGCG. Only EGCG upregulated UCP-2 and PPAR- γ genes.
Protective potential of epigallocatechin-3-gallate against benign prostatic hyperplasia in metabolic syndrome rats [122]	Chen et al., 2016	Rat models were fed a HFD for 12 weeks. Testosterone was injected at 10 mg/kg/d and EGCG was given orally for weeks 9–12	EGCG significantly decreased measured glucose levels, total cholesterol, triglycerides, IGFs, and inflammatory cytokines
The major green tea polyphenol, (-)-epigallocatechin-3-gallate, inhibits obesity, metabolic syndrome, and fatty liver disease in high-fat-fed mice [121]	Bose et al., 2008	Mice were fed a HFD and concurrently treated with EGCG supplementation or received no treatment for 16 weeks. Weight gain, percent body fat, and visceral fat were measured.	EGCG treated mice showed decreased insulin resistance, plasma cholesterol, and inflammatory cytokines.
EGCG reduces obesity and white adipose tissue gain partly through AMPK activation in mice [123]	Li et al., 2018	Mouse model: mice were fed a HFD for 20 weeks and 100 mg/kg EGCG was administered intragastrically/d. A control group fed a HFD, but no EGCG was present.	EGCG treatment group showed improved serum lipids, increased excretion of free fatty acids in feces, and decreased adipose tissue.

**Table 2:** Catechin and Antioxidant Human Studies.

Study Name	Author and Year	Methods	Result
The anti-obesity effects of green tea in human intervention and basic molecular studies [113]	Huang et al., 2014	Open, uncontrolled study in moderately obese population for 3 months	3.5 kg weight loss and 4.14 decrease in waist circumference in western populations
Effect of tea catechins with caffeine on energy expenditure in middle-aged men and women: a randomized, double-blind, placebo-controlled, crossover trial [132]	Katada et al., 2020	RCT, double-blind, crossover of 30 participants (mix of male and female) were given 611 mg or 0 mg catechins for 2 weeks. RMR and EE were measured.	EE was significantly increased in the treatment group as compared to the placebo. No significant difference in RMR was recorded.
The effects of polyphenol supplementation on adipose tissue morphology and gene expression in overweight and obese humans [133]	Most et al., 2018	RCT, 25 participants received either 282 mg/d EGCG, 80 mg/d RES or a placebo for 12 weeks. SAT was biopsied to access treatment effectivity.	Treatment group showed downregulation of cellular pathways contributing to adipogenesis and significantly decreased metabolic pathways that contribute to oxidative stress.
Diet supplementation with green tea extract epigallocatechin gallate prevents progression to glucose intolerance in db/db mice [120]	Ortsäter et al., 2012	RCT; 7-week old db/db mice received either EGCG, rosiglitazone or placebo for 10 weeks. Fasting glucose, body weight and food intake were measured during this time. Pancreata samples taken at end.	EGCG treatment group showed improved glucose tolerance, increased insulin secretion, and lowered the number of pathologically damaged β-pancreatic islet cells.
Efficacy of a green tea extract rich in catechin polyphenols and caffeine in increasing 24-h energy expenditure and fat oxidation in humans [134]	Dulloo et al., 1999	RCT; 10 males underwent 3 treatments (EGCG/caffeine, caffeine, placebo) on separate occasions. These treatments were orally ingested 3x/d.	Compared to placebo, the EGCG/caffeine treatment had a significant increase in 24 h EE and a significant decrease in respiratory quotient. Caffeine only treatment did not show these results as compared with placebo.
Effects of catechin enriched green tea on body composition [127]	Wang et al., 2010	RCT; moderately overweight Chinese subjects orally ingested various doses or EGCG 2 times/d. Data was collected at days 0, 30, 60, and 90.	The group with the highest dose of EGCG had a 5.6 cm^2^ decrease in intra-abdominal fact, a 1.9 cm waist circumference reduction, and a mean 1.2 kg body weight decrease.

## Abbreviations

AMPK	AMP-activated protein kinase
ATP	Adenosine triphosphate
BAT	Brown adipose tissue
BMI	Body mass index
CHOL	Cholesterol
COX	Cyclooxygenases
DNA	Deoxyribonucleic acid
EAT	Epididymal adipose tissue
EGC	Epigallocatechin
ECG	Epicatechin gallate
EE	Energy expenditure
EGCG	epigallocatechin gallate
ETC	Electronic chain transport
Gclc	Glutamate-cysteine ligase
GP	Glutathione peroxidase
H_2_O_2_	Hydrogen peroxide
HDL	High-density lipoproteins
HFD	High-fat diet
LDL	Low-density lipoproteins
LOX	Lipoxygenases
MAPK	Mitogen-activated protein kinase
MDA	Malondialdehyde
NADPH	Nicotinamide adenine dinucleotide phosphate
O_2_	Superoxide radicals
OH	Hydroxyl radicals
PK	Parkinson’s disease
PPAR	Proliferator-activated receptor gamma
PUMC	Pro-opiomelano cortin
RCT	Randomized clinical trial
RMR	Resting metabolic rate
ROS	Reactive oxidative stress
SAT	Subcutaneous adipose tissue
SOD	Superoxide dismutase
SVF	Stromal vascular fractions
TNFa	Tumor necrosis factor alpha
T2DM	Type 2 diabetes mellitus
U.S.	United States
VAT	Thermoplasma acidophilum
WAT	White adipose tissue
WD	Western diet
WHO	World Health Organization
XO	Xanthine oxidases
XDH	Xanthine dehydrogenase
XOD	Oxidoreductase
1O_2_	Singlet oxygen
